# Mitochondrial Changes Induced by SGLT2i in Lymphocytes from Diabetic Kidney Transplant Recipients: A Pilot Study

**DOI:** 10.3390/ijms26073351

**Published:** 2025-04-03

**Authors:** Isabel Pérez-Flores, Andrea R. López-Pastor, Ulises Gómez-Pinedo, Andrea Gómez-Infantes, Laura Espino-Paisán, Natividad Calvo Romero, M. Angeles Moreno de la Higuera, Beatriz Rodríguez-Cubillo, Irene Gómez-Delgado, Ana I. Sánchez-Fructuoso, Elena Urcelay

**Affiliations:** 1 Nephrology Department, Health Research Institute of Hospital Clínico San Carlos (IdISSC), Universidad Complutense de Madrid, 28040 Madrid, Spain; perezfloresi@yahoo.es (I.P.-F.); naticalvo75@gmail.com (N.C.R.); angie.moreno@gmail.com (M.A.M.d.l.H.); brcubillo@gmail.com (B.R.-C.); sanchezfructuoso@gmail.com (A.I.S.-F.); 2 Laboratory of Genetics and Molecular Bases of Complex Diseases, Health Research Institute of Hospital Clínico San Carlos (IdISSC), 28040 Madrid, Spain; andrea.raposo@salud.madrid.org (A.R.L.-P.); lauraep80@gmail.com (L.E.-P.); elena.urcelay@salud.madrid.org (E.U.); 3 Cooperative Research Networks Oriented to Health Results (RICORS, REI), 28089 Madrid, Spain; 4 Laboratory of Neurobiology and Advanced Therapy, Health Research Institute of Hospital Clínico San Carlos (IdISSC), Universidad Complutense de Madrid, 28040 Madrid, Spain; ulisesalfonso.gomez@salud.madrid.org; 5 Department of Medicine, Medical School, Universidad Complutense de Madrid, 28040 Madrid, Spain

**Keywords:** SGLT2i, kidney transplant, mitochondria, immune cells

## Abstract

Sodium-glucose co-transporter 2 inhibitors (SGLT2i) preserve cardiac and renal function by mechanisms that are not completely elucidated. Among other things, SGLT2i promote nutrient-deprivation signalling, which might affect the immune function. As the fate of immune cells is controlled by their metabolism, we aimed to study the mitochondrial integrity of lymphocytes isolated from renal transplant recipients with type 2 diabetes (T2D) upon SGLT2i therapy instauration and six-month follow up. In this real-world pilot study, the mitochondrial respiration of isolated peripheral blood mononuclear cells was monitored in a Seahorse XFp extracellular-flux analyzer and cells were photographed with a confocal microscope. Mitochondrial mass, membrane potential, and superoxide content of lymphocyte subpopulations were measured by flow cytometry (MitoTracker^TM^ Green, TMRM, and MitoSOX^TM^ Red probes). Leveraging in vivo conditions of immune cells, we evaluated their metabolic profiles associated with immune activation. Herein, we identified changes in redox homeostasis with sustained membrane polarization, and an increased mitochondrial biogenesis upon PHA stimulation that significantly correlated with changes in body weight and LDL-cholesterol levels, and a resultant compensatory mitochondrial function of lymphocytes. Our data suggest novel mechanisms induced by SGLT2i to modulate immune cells, which probably underlie the observed beneficial effects in kidney transplant recipients. Nonetheless, further mechanistic studies are required to extend these exploratory findings and encourage the use of this therapeutic strategy.

## 1. Introduction

Type 2 sodium-glucose cotransporter inhibitors (SGLT2i) reduce glucose reabsorption in the renal proximal tubules and thereby enhance urinary glucose excretion. The significant reduction in the risk of major adverse cardiovascular events and combined renal outcomes was originally identified through clinical studies in patients with type 2 diabetes (T2D) with cardiovascular disease (CVD) [1]. The renoprotective effects of SGLT2i were also observed in patients with a glomerular filtration rate (eGFR) below a threshold capable of yielding a glucose-lowering impact, which evidenced intrinsic protective effects of these agents on the kidney beyond the modest hypoglycemia, subsequently reported even in non-diabetic patients [2].

The SGLT2i role in improving renal function has been found to depend on pleiotropic actions, including suppression of inflammation and fibrosis, and enhanced renal oxygenation, but the underlying mechanisms are still not completely understood [3]. Processes such as natriuresis linked to blood pressure-lowering properties and changes in vascular function were initially considered. Nonetheless, clinical studies did not support the pathophysiological importance of these hemodynamic changes [4].

With the kidney being the organ with the second highest energetic demand [5], a mitochondrial impairment seems a plausible trigger to reduce renal functionality [6]. The role of SGLT2i in the preservation of mitochondrial function and cellular integrity contributes to maintaining organ structure and function [7]. Among the plethora of beneficial effects of SGLT2i, both decreased oxidative stress and inhibited proinflammatory pathways impact the evolution and progression of cardiomyopathy and nephropathy [8]. Nonetheless, direct changes in renal or cardiovascular physiology, energy use, or metabolism do not fully explain their benefits. Recently, patients treated with these glifozins have shown an increment in gluconeogenesis, ketogenesis, and decreased uricemia, and these distinctive effects evidenced a critical involvement of the balance between nutrient deprivation and nutrient surplus signalling [9].

The leading cause of chronic kidney disease (CKD) is T2D, a condition with a global prevalence of over 10% [10], and additionally, kidney transplant recipients show an increased risk for post-transplant T2D [11]. Renal transplantation is the preferred treatment for patients with end-stage disease, and transplant recipients often face unique challenges involving glucose metabolism and mitochondrial function, related to immunosuppressive therapy [12]. Growing evidence describes the benefits obtained by SGLT2i administration in T2D, CVD and CKD, and recently in kidney transplants [13]. However, the knowledge about SGLT2i mechanisms is currently incomplete [14,15]. The predominant SGLT2 expression in the proximal renal tubule [16] was initially the focus of study of these inhibitors directly on the kidney, providing data about the inflamed tissue hypoxia, acidosis, presence of specific cytokines, and high levels of metabolites derived from anaerobic glycolysis [17,18]. In addition, damaged endothelial cells enhance vascular permeability, with an expression of adhesion molecules and leukocyte recruitment and migration [19,20]. Consequently, we postulate that an immune modulation could be part of the systemic benefit provided by these agents [21]. Inflammation and oxidative stress [22] play a key role in the development and progression of renal dysfunction, with peripheral blood mononuclear cells (PBMCs) closely involved. Metabolic reprogramming of immune cells occurs in response to changes in the tissue microenvironment, as part of a balanced immune response [23].

In the current pilot study, the primary objective was to explore the impact of SGLT2i on the mitochondrial activity of PBMCs from kidney transplant recipients. Specifically, we expected to unravel changes in mitochondrial ROS, membrane depolarization, and mass in resting and activated PBMCs from transplanted individuals over a period of six months upon SGLT2i administration, as we propose that the mitochondrial response of immune cells might identify an underlying mechanism related to their renoprotective effects, probably with a crucial impact in these patients. As secondary objective, we evaluated the clinical benefits evidenced in other renal disorders. Our approach fully captures in vivo conditions and contributes to understand the role of immune cells and to ascertain new properties of these agents.

## 2. Results

### 2.1. Description of the Studied Cohort

Fourteen kidney transplant recipients with T2D, diagnosed according to the American Diabetes Association’s criteria, were enrolled in the study (see Table 1 and Table 2 for demographic and clinical variables, respectively), prescribed with a SGLT2i, and followed for at least 6 months to pursue a beneficial effect of this drug in their condition. No changes in other hypoglycemic drugs during the 6-month follow-up period under SGLT2i treatment were observed. The anthropometric and biochemical parameters measured were body weight, systolic (SBP) and diastolic (DBP) blood pressure, estimated glomerular filtration rate, urinary protein–creatinine ratio, haemoglobin level, glycaemia (fasting plasma glucose, HbA1c), lipid metabolism (serum triglycerides and total cholesterol), serum potassium, magnesium, and uric acid (Table 2). No significant changes were observed in glycated hemoglobin levels. Body weight and serum levels of magnesium significantly changed between baseline levels before SGLT2i therapy and tthree/six months after SGLT2i treatment (Table 2), and a sustained decrease in serum uric acid levels was also recorded.

### 2.2. Upon PHA-Stimulation, SGLT2i Treatment Induces an Increase in Mitochondrial Mass and Membrane Potential, but Not in ROS Content

To evaluate putative perturbations in mitochondrial activity derived of SGLT2i treatment, we aimed to assess potential changes in mitochondrial mass, membrane potential, and levels of reactive oxygen species (ROS) in PBMCs isolated from kidney transplant recipients.

The temporal evolution of mitochondrial mass in overall PBMCs and lymphocyte subpopulations in both unstimulated and PHA-stimulated conditions was explored, and significant reductions were consistently found in the unstimulated situation as well as correlative increases in the PHA^+^/PHA^−^ ratios (Table 3).

**Figure 1:**
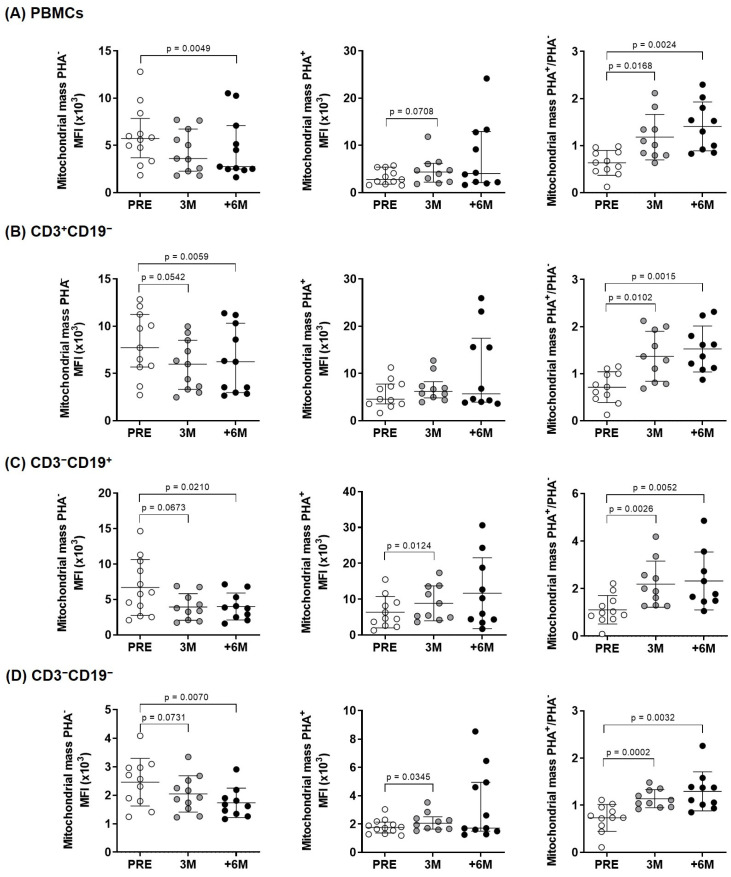
Mitochondrial mass in lymphocyte subpopulations of kidney-transplant recipients. Diagrams display unstimulated, PHA-stimulated, and fold increase in mitochondrial mass upon PHA-stimulation measured by flow cytometry (MitoTracker^TM^ Green FM probe) in (**A**) overall PBMCs and (**B**–**D**) lymphocyte subpopulations from the experimental groups (n = 9–11). Results were evaluated by paired two-tailed Student’s *t*-tests or by paired non-parametric Wilcoxon tests in groups that show no normal data with the Shapiro–Wilk test [in PHA^–^: +6M PBMCs and +6M CD3^+^CD19^−^; and in PHA^+^: +6M PBMCs, +6M CD3^+^CD19^−^, and +6M CD3^−^CD19^−^] (error bars indicate mean ± SD or median ± interquartile range, respectively). MFI: Median fluorescence intensity.

When comparing the situation before SGLT2i treatment (PRE) with three (3M) and six or more months (+6M) after its administration, in overall PBMCs (Figure 1A) and in the cell subpopulations studied (T- and B-cells and natural killer cells) (Figure 1B–D, respectively), we found statistically significant mitochondrial mass increases after cell activation (ratios PHA^+^/PHA^−^).

**Figure 2:**
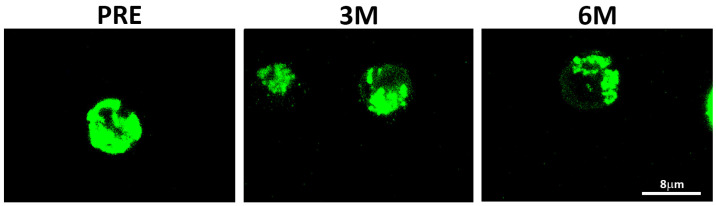
Mitochondrial distribution assessed by confocal microscopy in PBMCs of a representative subject. MitoTracker^TM^ Green FM probe detected before (PRE) and upon 3-month (3M) and 6-month or more (+6M) under SGLT2i treatment.

The normalized ratio of median fluorescence intensities (MFI signal) between PHA-stimulated and unstimulated sample pairs (PHA^+^/PHA^−^ ratios) quantified net increases in mitochondrial mass. In basal unstimulated conditions, significant differences corresponded mainly to a progressive decrease in mitochondrial mass upon SGLT2i treatment, as visualized by confocal microscopy (Figure 2).

The observed changes in mitochondrial mass could have an impact in clinical variables, and we identified an inverse correlation with Hba1c specifically in the PHA-stimulated condition, which reached statistical significance in overall PBMCs, and the same trend was observed in the different subpopulations studied (Table 4). The normalized changes of mitochondrial mass upon PHA stimulation showed direct correlations with body weight and inverse correlations with total cholesterol, mainly driven by LDL content, in each lymphocyte subpopulation (Table 4).

**Figure 3:**
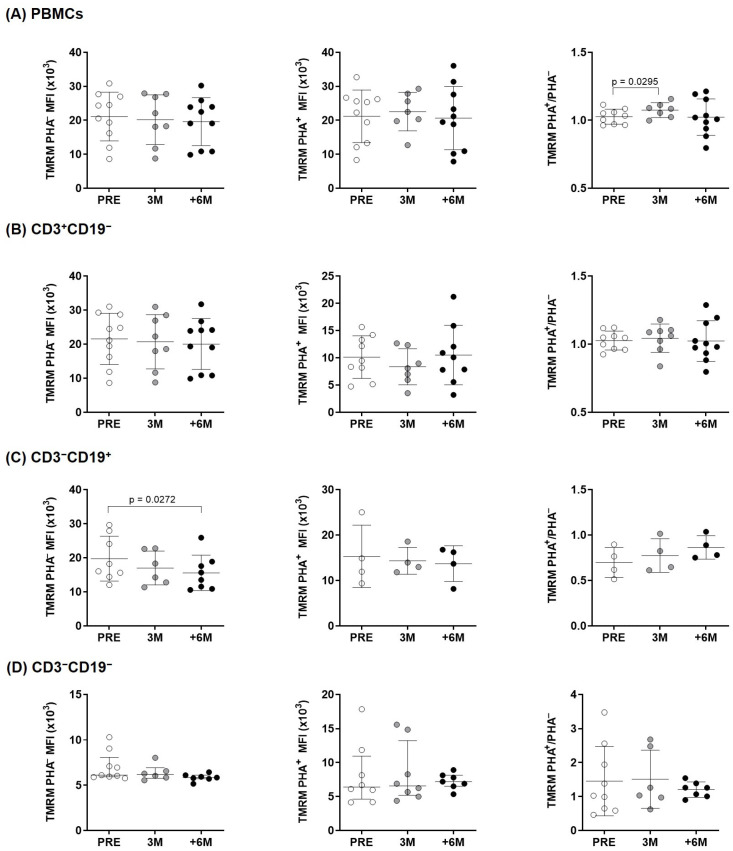
Mitochondrial membrane potential changes in lymphocytes of kidney transplant recipients. Mitochondrial membrane potential before and upon PHA-stimulation, measured by flow cytometry (MitoProbe^TM^ TMRM) in (**A**) overall PBMCs and (**B**–**D**) lymphocyte subpopulations from the experimental groups (n = 4–10). Statistical significance assessed by paired two-tailed Student’s *t*-tests and by paired non-parametric Wilcoxon tests in groups that show no normal data with the Shapiro–Wilk test [in PHA^–^: PRE CD3^−^CD19^−^; and in PHA^+^: 3M CD3^−^CD19^−^] (error bars indicate mean ± SD or median ± interquartile range, respectively). MFI: Median fluorescence intensity.

We next used TMRM to assess mitochondrial membrane potential across the inner mitochondrial membrane, as a readout of the movement of protons and functionality of the electron transport chain, ETC (Figure 3). Significant changes were observed only in B cells treated for 6M in basal conditions and for the normalized PHA^+^/PHA^−^ ratio in overall PBMCs upon 3M SGLT2i therapy when compared to the pre-treatment situation.

**Figure 4:**
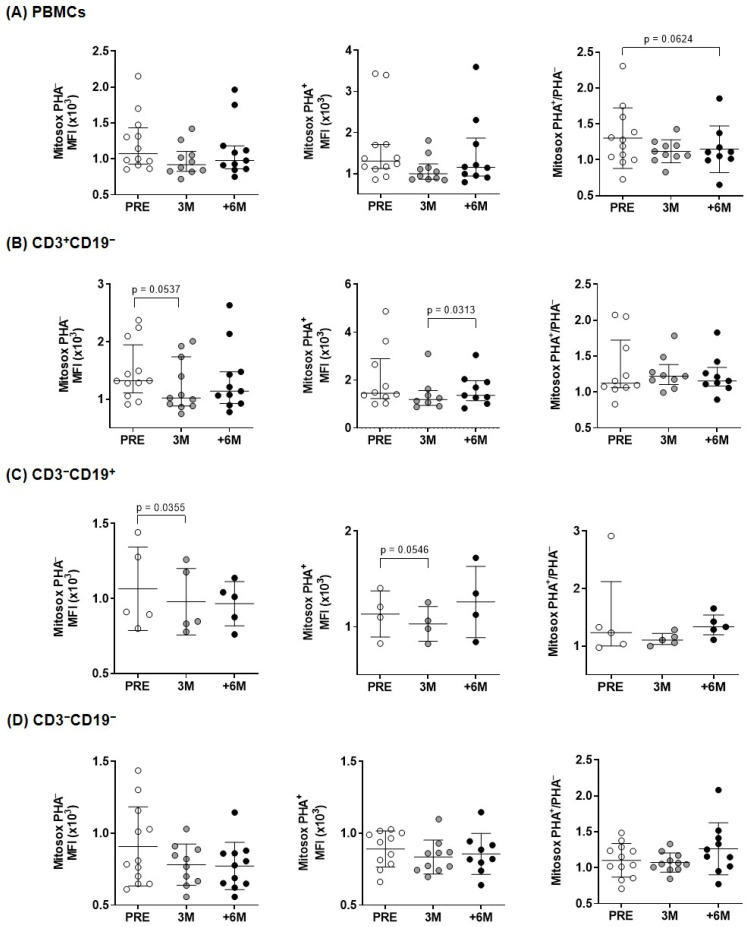
Mitochondrial superoxide content in lymphocytes of kidney transplant recipients treated with SGLT2i. Mitochondrial superoxide anion levels produced before and after PHA-stimulation, measured by flow cytometry with MitoSOX^TM^ Red reagent for: (**A**) overall PBMCs and (**B**–**D**) lymphocyte subpopulations from kidney recipients (n = 4–11). Statistical significance assessed by paired two-tailed Student’s *t*-test and by paired non-parametric Wilcoxon test in groups that show no normal data with the Shapiro–Wilk test [PHA^–^ (PRE and +6M PBMCs, 3M and +6M CD3^+^CD19^−^), PHA^+^ (all conditions in PBMCs, PRE and 3M CD3^+^CD19^−^), and PHA^+^/PHA^–^ (CD3^+^CD19^−^ and CD3^−^CD19^+^)]. (error bars indicate mean ± SD or median ± interquartile range, respectively).

Since mitochondrial dysfunction is highly associated with the production of ROS, the expression of mitochondrial ROS was investigated in overall PBMCs and in the different lymphocyte subpopulations (Figure 4). The CD3^+^CD19^−^ and CD3^−^CD19^+^ subpopulations (T- and B-lymphocytes) showed a significantly lower content of superoxide production after 3 months of treatment, and similar trends to decrease after SGLT2i treatment were found upon PHA-stimulation (Figure 4). In overall PBMCs, the normalized PHA^+^/PHA^−^ ratio evidenced a borderline significant ROS decline upon 6M follow up (Figure 4).

### 2.3. Mitochondrial Activity After SGLT2i Therapy

**Figure 5:**
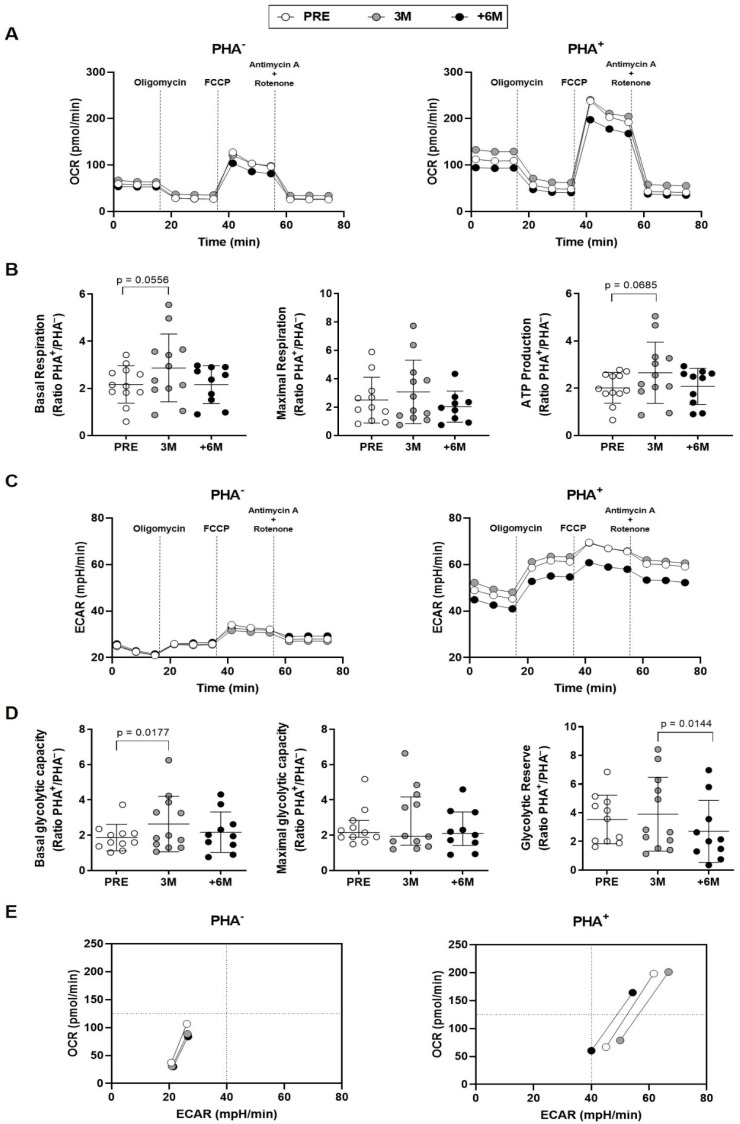
Effect of SGLT2i treatment on lymphocyte mitochondrial activity in kidney transplant recipients. (**A**) Mitochondrial OCR profiles of unstimulated and PHA-stimulated PBMCs from patients (n = 12) prior (PRE), after 3 months, and after 6 or more months (+6M) with SGLT2i treatment (traces represent means). (**B**) Mitochondrial parameters shown as fold increase upon PHA-stimulation. (**C**) Mitochondrial ECAR profiles of unstimulated and PHA-stimulated PBMCs. (**D**) Mitochondrial parameters presented as fold increase upon PHA-stimulation. (**E**) Energy phenotypes of PBMCs (points represent means of basal, maximal respiration and glycolytic capacity). Statistical significance assessed by two-tailed Student’s *t*-tests (error bars: means ± SD) and non-parametric Wilcoxon tests for maximal glycolytic capacity (error bars: median ± interquartile range).

The observed changes in mitochondria of lymphocytes from kidney transplant recipients with SGLT2i treatment prompted us to further study the mitochondrial functional status of those PBMCs in both basal and PHA-stimulated conditions (Figure 5).

The oxygen consumption rates kinetics (OCRs) in PHA-stimulated PBMCs from individuals treated with SGLT2i over 6 months showed a decreased profile in comparison with either the situation found prior to the administration of the inhibitor or after 3 months of treatment (Figure 5A, right panel). When aerobic respiration parameters were disclosed, a statistically significant fold increase upon PHA-stimulation between untreated and 3M treatment was detected for basal respiration, and a similar trend was observed for ATP production (Figure 5B).

The extracellular acidification rate (ECAR) kinetics in unstimulated conditions evidenced similar patterns in lymphocytes of kidney recipients along the six months of SGLT2i therapy (Figure 5C, left panel). Upon PHA stimulation, PBMCs from the group with +6-month SGLT2i treatment also showed a lower ECAR profile in comparison with the untreated and 3M groups (Figure 5C, right panel). After three months of SGLT2i treatment, the fold change in the presence and absence of PHA proved a significant increase in basal glycolytic capacity (Figure 5D). Regarding the estimation of glycolytic reserve, we observed statistically significant lower levels after +6-month of SGLT2i treatment when compared to 3M (Figure 5D).

These changes were summarized in the energy phenotypes, which represent the simultaneous comparison of mitochondrial respiration and extracellular acidification under baseline and stressed conditions, to define the metabolic potential in response to an energy demand. Minimum and maximum values of OCR and ECAR allowed an estimation of the metabolic range of the cells. The energy phenotypes in basal conditions (Figure 5E, left panel) uncovered a slightly less aerobic status for the different extents of treatment. Upon PHA stimulation (Figure 5E, right panel), the temporal evolution towards a more quiescent situation displayed first a transitory higher glycolysis observed already after 3M therapy; then, after +6M, less glycemic levels and a lower aerobic rank were reached.

## 3. Discussion

SGLT2 inhibition at proximal tubules leads to glucosuria and natriuresis. The reduced sodium reabsorption decreases intraglomerular pressure, glomerular hyperfiltration and albuminuria due to restoration of the tubulo-glomerular feedback system. Originally, this mechanism was suggested to be responsible for the renoprotective effects of SGLT2i [24], but this is only part of the picture [25]. Concordantly, our results do not show major changes in GFR or uPCR upon SGLT2i treatment (Table 2). Other parameters reflect already described [26] modifications indicative of improved renal homeostasis, such as the progressive drop of serum uric acid [27], the significant increase in serum magnesium, and some body weight loss (Table 2).

Preclinical studies evidenced the effect of these drugs on mitochondrial function, which contributed to their renal and cardiac benefits [28,29]. Our results support that SGLT2i do not only offer protective effects directly on both cardiac and kidney tissues, but they also improve PBMCs energetics. Immune cells are key players in metabolic diseases, specifically in T2D [30]. T2D patients show increased circulating proinflammatory cytokines and decreased anti-inflammatory IL-10 [31]. In this scenario, circulating leukocytes maintain a chronic low-grade inflammatory state and, to meet the metabolic demands for effector function, they switch from oxidative phosphorylation to aerobic glycolysis [23,32,33]. These demands require an adequate mitochondrial activity, as seemingly supported by SGLT2i treatment in PBMCs isolated from kidney transplant patients. Following six months of treatment, we observe a net increase in mitochondrial mass upon PHA stimulation in overall lymphocytes and consistently in each subcellular population (Figure 1). As shown, these changes reflect mainly the decreasing mitochondrial mass in unstimulated conditions and inverse changes under PHA stimulation. In physiological conditions, mitochondria continuously fuse and divide in a highly regulated balance, and damaged depolarized mitochondria are removed via mitophagy to preserve cellular homeostasis [34]. Herein, the normalized mitochondrial mass of immune cells increases as treatment progresses, which seems indicative of their systemic SGLT2i beneficial properties. The observed changes in membrane potential, with significant lower levels in basal conditions only in B cells (Figure 3), mirror those found in mitochondrial mass; nonetheless, the normalized ratios only showed a transient significant difference after 3 months of treatment in overall lymphocytes, with no other significant changes detected in the membrane potential of the studied lymphocyte subpopulations.

Under physiologic conditions, antioxidant mechanisms balance ROS produced during ATP synthesis. Oxidative stress appears when ROS generation surpasses local antioxidant capacity and causes oxidative damage to mitochondria. Therefore, ROS content is a critical indicator of overall mitochondrial function. As shown in Figure 4 (left panels), we observed a reduction in mitochondrial superoxide production in PBMCs and the different subpopulations from transplanted patients after treatment with SGLT2i, in agreement with similar results that previously reported lower mitochondrial superoxide in leukocytes of diabetic patients [35,36], in cell lines [37] and also in diabetic rats [38]. Similarly, after myocardial infarction, SGLT2i prevented diabetes-induced excessive reduction in mitochondrial size via suppression of ROS [39]. 

As shown, the increased mitochondrial mass upon PHA stimulation (Figure 1, PHA^+^/PHA^−^ ratios) with a sustained membrane potential (Figure 3) preclude a dysfunctional mitochondrial and support the preserved efficiency of the ETC. These data prompted us to delve deep into the mitochondrial activity of immune cells isolated from our patients.

In a healthy kidney, the main energetic source rises via oxidative phosphorylation. In the presence of diabetic kidney disease, the hyperglycemia and consequent hyperfiltration increase the energy requirements and the resulting hypoxia shifts metabolism from oxidative phosphorylation to glycolysis, less efficient for energy production [40,41]. The imbalance between decreased production and increased demand causes an overall energetic deficiency. Organ-based studies found that SGLT2i promote a nutrient-deprivation signalling and help restoring mitochondrial function [42]. These inhibitors increase nutrient oxidation and oxidative phosphorylation, and reduce the cytosolic accumulation of deleterious glucose and lipid by-products. Our results indicate that, in addition to the improved renal energy production described for SGLT2i, their effects extend to lymphocyte energetics where we acknowledge significant changes. Some of them seem transitory (detected at 3M, Figure 5E), and ultimately reflect a compensatory mechanism mainly in response to an immune activator, PHA. These SGLT2i-mediated protective effects include a final reduction in oxygen consumption as shown in the energetic profile, parallel to the one reported in diabetic kidney [17,43], and a decrease in glycolysis.

Although with limited sample size, our pilot study provides valuable evidence of the immune cells properties of SGLT2i treatment in kidney transplant subjects, in line with recently found circulating protein signatures driven by SGLT2i, which demonstrate a key role of mitochondrial proteins [44] and of oxidative stress modulation genes [45,46,47]. Concordantly, a selective regulation of immune responses by targeting specific metabolic pathways was formerly associated with health benefits in transplant [48]. As our results show, alternative SGLT2i mechanisms include responses that go beyond the direct effects on kidney and seem to compensate a possible mitochondrial dysfunction of immune cells in diabetic renal transplant recipients. These additional SGLT2i actions would alleviate the mitochondrial stress potentially present in lymphocytes of diabetic kidney recipients, and would lead to a metabolically quiescent state aimed to the conservation of cellular functions. In fact, improved mitochondrial oxidative stress and biogenesis-related pathways are shared processes with other T2D drugs, such as glucagon-like 1 peptide receptor agonists (GLP-1RA), and both warrant further mechanistic research [49,50]. Moreover, despite the known ethnic differences in the phenotype and pathophysiology of T2D, the clinical efficacy of SGLT2i as an add-on treatment to metformin monotherapy is similar in Asian and non-Asian T2D patients [51]. Nonetheless, this point deserves specific research in other regimens and conditions.

In summary, our results in diabetic kidney-transplanted individuals support the use of SGLT2 inhibitors [52]. Their functional benefits transcend those solely explained by glycemic control and point to the preservation of mitochondrial activity in lymphocytes. In addition to other cardio and renoprotective actions, the described mitochondrial adaptation of immune cells to an activation signal might support an immunomodulatory function of this therapy [32] that would help restoring homeostasis.

## 4. Materials and Methods

### 4.1. Study Population

We conducted an observational prospective pilot study in accordance with the Strengthening the Reporting of Observational Studies in Epidemiology (STROBE) guidelines [53] (Supplementary Materials, Table S1). The patient recruitment for this study was conducted between January 2021 and July 2022 at Hospital Clinico San Carlos (Madrid, Spain). The study population consisted of adults aged 50 to 80 years who underwent renal transplant and were prescribed SGLT2i for the adequate glycemic control of their T2D. Inclusion criteria: (a) adult (over 18 y.o.); (b) recipients of kidney transplants at our center; (c) diagnosed prior or post-transplantation with T2D; (d) prescribed a SGLT2i to support an adequate glycemic control. Exclusion criteria: (a) changes in the hypoglycemic treatment during the 6-month follow-up with SGLT2i, (b) drop of SGLT2i treatment during the 6 month follow up, because of either lack of adherence of the patient or suspension due to side effects by the prescribing physician. The initial study included 30 subjects, but only 14 (35.7% females) were finally enrolled, as most participants met one or more exclusion criteria.

All participants were included after informed consent. The study was approved by the Ethics Committee of Hospital Clínico San Carlos (Madrid, Spain). Demographic, clinical, and laboratory (glomerular filtration rate (GFR), proteinuria, and different biochemical parameters) data of each individual were extracted from electronic medical records (Table 1 and Table 2).

### 4.2. Sample Collection

Peripheral blood samples were collected in EDTA tubes before (PRE), three (3M) and six or more months (+6M) after the establishment SGLT2i treatment. They were transferred into Vacutainer CPT Mononuclear Cell Preparation tubes (362782, BD Biosciences, Franklin Lakes, NJ, USA) to isolate peripheral blood mononuclear cells (PBMCs) (30 min/1200× *g* at room temperature) and washed with Hank’s balanced salt solution (21-022-CV, Corning, Corning, NY, USA). Subsequently, 10^7^ PBMCs in freezing medium {fetal bovine serum (F9665, Sigma Aldrich, St. Louis, MO, USA) with 10% dimethylsulfoxide (DMSO, LM-0102-M010.0-005, Biotech, Berlin, Germany)} were cryopreserved until further analysis.

### 4.3. Cell Metabolism

Oxygen consumption rate (OCR) and extracellular acidification rate (ECAR) of isolated PBMCs from individuals under SGLT2i treatment were simultaneously monitored in a Seahorse XFp extracellular flux analyzer (Agilent Technologies, Santa Clara, CA, USA). First, PBMCs were counted after thawing to seed 1.5 × 10^6^ cells per mL in RPMI 1640 (15-041-CV, Corning, Corning, NY, USA). The next day, another cell counting was carried out to perform the stimulation. Specifically, for each participant, one half of the cells were stimulated with 5 μg/mL phytohemagglutinin (PHA, L1668, Sigma Aldrich, St. Louis, MO, USA) for 24 h, to assess the metabolism of activated PBMCs, and the other half left without stimulation to test basal metabolism [54]. After 24 h of PHA-stimulation, a rigorous final cell count (6 times per experimental group) was performed, and split in triplicates with 2 × 10^5^ PBMCs per well seeded onto poly-D-lysine coated XFp plates (103022-100, Agilent Technologies, Santa Clara, CA, USA), which allowed to certify reproducibility. Upon a non-carbonated incubation for 50 min at 37 °C, basal and PHA-stimulated conditions were assayed in a Seahorse run for 70 min in XF DMEM medium, pH 7.4 (103575-100, Agilent Technologies, Santa Clara, CA, USA), supplemented with glucose (10 mM), glutamine (2 mM), and pyruvate (1 mM). Then, mitochondrial and glycolytic functions were assessed with the Cell MitoStress Test kit (103010-100, Agilent Technologies, Santa Clara, CA, USA), following manufacturer’s instructions. Briefly, cells were sequentially treated with mitochondrial oxidative phosphorylation (OXPHOS) selective inhibitors: 1.5 μM oligomycin to inhibit ATP synthase and evaluate the ATP production efficiency of the cells; 0.5 μM FCCP (carbonylcyanide-p-trifluoromethoxyphenylhydrazone) which uncoupled mitochondrial respiration to determine the maximal substrate oxidation capacity; and a cocktail of rotenone and antimycin A at 0.5 μM to inhibit mitochondrial respiration and evaluate non-mitochondrial OCR. Agilent Seahorse Wave analyzed these data, with each parameter calculated as follows: Basal Respiration: (Last OCR measurement before oligomycin injection)–(Non-Mitochondrial Respiration). Maximal Respiration: (Maximum OCR measurement after FCCP injection)–(Non-Mitochondrial Respiration). ATP Production: (Last OCR measurement before Oligomycin injection)–(Minimum OCR measurement after Oligomycin injection). Basal Glycolytic Capacity: Last ECAR measurement before Oligomycin injection. Maximal Glycolytic Capacity: Maximum ECAR measurement after FCCP injection.

### 4.4. Flow Cytometry

To detect lymphocyte subpopulations {CD3^+^CD19^−^ (T lymphocytes), CD3^−^CD19^+^ (B lymphocytes), and CD3^−^CD19^−^ (mostly natural killer (NK) cells)}, 2 × 10^5^ PBMCs were stained with anti-CD3-PE or anti-CD3-APC/Fire^TM^ and anti-CD19-APC or anti-CD19-PB (BioLegend, San Diego, CA, USA). Mitochondrial mass, membrane potential, and superoxide content of PBMCs (MitoTracker^TM^ Green FM probe, M46750; MitoProbe^TM^ TMRM, M20036; MitoSOX^TM^ Red reagent, M36008, Invitrogen, Waltham, MA, USA) were measured following manufacturer’s instructions. Specifically, MitoTracker^TM^ Green FM probe was added to PBMCs incubated in RPMI 1640 (15-041-CV, Corning, Corning, NY, USA) to a final concentration of 1 mM/30 min/37 °C; in MitoProbe^TM^ TMRM assay, the final concentration was 20 nM/30 min/37 °C; alike, MitoSOX^TM^ Red reagent in a final concentration of 3.8 µg/µL was incubated for 15 min. Upon incubation of each probe, we performed a centrifugation to remove RPMI. Samples were also stained with 7-aminoactinomycin D (7-AAD) to exclude non-viable cells in MitoTracker^TM^ Green FM and MitoProbe^TM^ TMRM assays (A07704, Beckman Coulter, Brea, CA, USA).

Gating strategies (CytoFLEX cytometer, Beckman Coulter, Brea, CA, USA) with antibodies used according to the manufacturer’s instructions (Supplementary Materials, Table S2) are presented in Figures S1–S3 (Supplementary Materials). Data were analyzed with Kaluza v2.1. software (Beckman Coulter, Brea, CA, USA).

### 4.5. Confocal Microscopy

PBMCs of three patients in each time point (PRE, 3M and +6M) were stained and incubated with MitoTracker^TM^ Green FM probe (M46750, Invitrogen, Waltham, MA, USA) and DAPI (D9542, Sigma Aldrich, St. Louis, MO, USA) for 30 min at 37 °C. PBMCs were seeded at 7.5 × 10^4^ cells per well onto poly-D-lysine (1:5000, A38904-01, Gibco, Waltham, MA, USA) coated-bottom glass dishes (D35-10-1.5-N, Cellvis, Sunnyvale, CA, USA). Subsequently, coated dishes were photographed with an Olympus FV3000 Confocal Laser Scanning Microscope (Olympus Corporation, Tokio, Japan).

### 4.6. Statistical Analysis

The normality of variables was assessed with Shapiro–Wilk test (less than 30 data). Outliers were detected and discarded with Grubbs’ test (https://www.graphpad.com/quickcalcs/Grubbs1.cfm, GraphPad online tool, accessed on 20 July 2024). Differences among times were assessed using paired one-way ANOVA tests or paired two-tailed Student’s *t*-tests, and paired non-parametric Wilcoxon tests. Parametric data were expressed as mean ± standard deviation (SD), and non-parametric data as a median with interquartile range (IQR). Qualitative parameters (sex and ethnicity) were analysed by Pearson’s chi-squared test. Correlation analyses were performed using Spearman’s rank correlation tests. A *p* < 0.05 was considered statistically significant. SPSS v.15.0.1. (SPSS Inc., Chicago, IL, USA) and Prism v8.0. (GraphPad Software Inc., San Diego, CA, USA) allowed the analyses and graphical representations. Initially, 14 patients comply with the inclusion and follow up criteria. Nonetheless, sometimes a smaller number is considered for statistical analysis of each outcome when the amount of a biological sample is limited to complete every determination, when individual differences in lymphocyte subpopulations hamper reaching the established threshold of positive events in flow cytometry (200 × 10^3^ cells), or else when statistical outliers are detected.

## 5. Conclusions

These results demonstrate that a six-month treatment with SGLT2i significantly lowers body weight and increases serum magnesium in a novel target population: diabetic kidney transplanted patients. Serum uric acid levels showed a substantial reduction, in agreement with previously described benefits of SGLT2 inhibitors. Other clinical parameters reportedly influenced by these drugs were already normalized in our patients when treatment began. Despite the limited sample size, our study evidences an increased mitochondrial mass and reduced superoxide content with preserved mitochondrial potential in lymphocytes upon PHA stimulation, indicative of an immune modulatory role that supports the efficacy of SGLT2i in managing diabetic kidney-transplanted individuals. Further research is needed to fully explain the underlying mechanisms of these inhibitors and to expand their use.

## Figures and Tables

**Table 1:** Demographic data of kidney transplant patients (n = 14) upon recruitment for SGLT2i treatment. Results are expressed as mean (standard deviation, SD), n (%) or median (interquartile rate, IQR).

Age [Years, Mean (SD)]	66.4 (10.2)
Male, n (%)	10 (71.4)
Months after transplantation, median (IQR)	103 (48–205)
Pre-transplant T2D, n (%)	4 (28.6)
Pre-transplant T2D duration [years, median (IQR)]	18 (12.9–36.1)
Post-transplant T2D, n (%)	10 (71.4)
Post-transplant T2D duration [months, median (IQR)]	54.7 (16.8–146.9)
Immunosuppression Therapy, n (%)	
Tacrolimus	12 (85.7)
Sirolimus	3 (21.4)
MPA (Mycophenolic acid)	8 (57.1)
Steroids	5 (35.7)
Antidiabetic agents, n (%)	
Insulin	4 (28.6)
Metformin	4 (28.6)
Insulin and Metformin	2 (14.3)
Non-antidiabetic agents	4 (28.6)
SGLT2i, n (%)	
Dapaglifozin	11 (78.6)
Empagliglozin	3 (21.4)

**Table 2:** Evolution of clinical variables in renal transplant patients under treatment with SGLT2i. Results expressed as mean (standard deviation, SD), except for uPCR expressed as median (interquartile rate, IQR). Statistical significance evaluated by one-way ANOVA test to compare PRE vs. −3 and −6 months (p1) and PRE vs. +3 and +6 months (p2). eGFR: estimated glomerular filtration rate; SBP: systolic blood pressure; uPCR: urinary protein/creatinine ratio.

	−6M	−3M	PRE	+3M	+6M	p1	p2
eGFR (mL/min)	51.5 (25.9)	48.1 (20.7)	44.9 (16.6)	43.7 (14.5)	43.1 (16.6)	0.212	0.784
uPCR (mg/mg)	0.20 (0.14–0.48)	0.18 (0.12–1.00)	0.22 (0.11–0.57)	0.33 (0.12–0.56)	0.21 (0.19–0.49)	0.723	0.212
Fast glycaemia (mg/dL)	137 (48)	134 (36)	130 (30)	124 (35)	131 (28)	0.560	0.863
HbA1c (%)	6.6 (0.8)	6.4 (0.5)	6.9 (0.8)	6.6 (0.6)	6.7 (0.9)	0.051	0.305
Total cholesterol (mg/dL)	156 (40)	148 (22)	145 (32)	149 (33)	137 (37)	0.508	0.501
Triglycerides (mg/dL)	147 (30)	214 (103)	173 (48)	149 (50)	179 (88)	0.270	0.460
Body weight (kg)	78.0 (23.4)	82.5 (11.5)	80.9 (15.6)	79 (15)	77.4 (15.0)	0.058	**0.002**
SBP (mmHg)	130 (14)	126 (14)	136 (16)	134 (17)	130 (14)	0.079	0.651
Haemoglobin (g/dL)	13.4 (1.1)	13.5 (2.3)	13.4 (1.7)	13.5 (1.2)	13.5 (1.7)	0.555	0.732
Serum potassium (mEq/L)	4.4 (0.7)	4.1 (0.3)	4.1 (0.6)	4.2 (0.3)	4.3 (0.3)	0.371	0.298
Serum magnesium (mg/dL)	1.6 (0.3)	1.7 (0.1)	1.6 (0.2)	1.9 (0.2)	1.9 (0.2)	0.711	**0.004**
Serum uric acid (mg/dL)	6.7 (1.4)	6.2 (1.0)	6.2 (1.5)	6.0 (1.5)	5.7 (1.4)	0.357	0.157

**Table 3:** Evolution of mitochondrial mass in PBMCs and lymphocyte subpopulations in unstimulated and PHA-stimulated conditions. Results expressed as median mitochondrial mass (interquartile rate, IQR). Statistical significance evaluated by paired non-parametric Wilcoxon tests.

	PRE	3M	+6M	*p*
PBMCs PHA^−^	5716 (3698–7862)	3603 (2263–6731)	2753 (2496–7089)	**0.045**
PBMCs PHA^+^	2791 (1780–5385)	4354 (2207–6178)	4037 (2147–12,930)	0.717
PBMCs PHA^+^/PHA^−^	0.64 (0.45–0.86)	1.05 (0.79–1.46)	1.41 (0.90–1.85)	**0.013**
CD3^+^CD19^−^ PHA^−^	7735 (5682–11,262)	5994 (3321–8523)	6244 (2981–10,323)	**0.050**
CD3^+^CD19^−^ PHA^+^	4569 (3557–7760)	6211 (4825–8254)	5696 (3888–17,468)	0.895
CD3^+^CD19^−^ PHA^+^/PHA^−^	0.72 (0.46–1.00)	1.32 (0.80–1.95)	1.49 (1.11–1.91)	**0.004**
CD3^−^CD19^+^ PHA^−^	5896 (3021–10,076)	3792 (2114–6765)	3816 (2400–5699)	**0.045**
CD3^−^CD19^+^ PHA^+^	4641 (2623–9087)	7157 (4753–13,607)	8115 (4114–20,177)	0.121
CD3^−^CD19^+^ PHA^+^/PHA^−^	1.01 (0.75–1.82)	1.94 (1.29–2.76)	1.76 (1.48–2.94)	**0.004**
CD3^−^CD19^−^ PHA^−^	2566 (1792–2971)	1922 (1536–2517)	1648 (1326–1973)	0.169
CD3^−^CD19^−^ PHA^+^	1756 (1380–2133)	2053 (1648–2512)	1701 (1439–4692)	0.368
CD3^−^CD19^−^ PHA^+^/PHA^−^	0.75 (0.60–0.97)	1.08 (0.96–1.32)	1.24 (0.99–1.43)	**0.005**

**Table 4:** Correlations between mitochondrial mass and clinical variables in PBMCs and lymphocyte subpopulations under basal and PHA-stimulated conditions. Spearman’s rank correlation coefficient test was used to assess the degree of linear correlation. r: Spearman’s Rho.

Mitochondrial Mass in:	Hba1c	Body Weight	Total Cholesterol	LDL Cholesterol	HDL Cholesterol
PBMCs PHA^−^	r = −0.070 *p* = 0.828	r = −0.552 *p* = 0.063	r = 0.300 *p* = 0.370	r = 0.231 *p* = 0.471	r = 0.238 *p* = 0.456
PBMCs PHA^+^	r = −0.728 ***p* = 0.011**	r = 0.173 *p* = 0.612	r = −0.536 *p* = 0.089	r = −0.727 ***p* = 0.011**	r = −0.018 *p* = 0.958
PBMCs PHA^+^/PHA^−^	r = 0.228 *p* = 0.475	r = 0.764 ***p* = 0.006**	r = −0.773 ***p* = 0.005**	r = −0.891 ***p* < 0.001**	r = −0.224 *p* = 0.484
CD3^+^CD19^−^ PHA^−^	r = −0.229 *p* = 0.499	r = −0.400 *p* = 0.223	r = 0.188 *p* = 0.603	r = 0.073 *p* = 0.832	r = 0.328 *p* = 0.325
CD3^+^CD19^−^ PHA^+^	r = −0.563 *p* = 0.071	r = 0.291 *p* = 0.385	r = −0.382 *p* = 0.247	r = −0.518 *p* = 0.102	r = −0.173 *p* = 0.612
CD3^+^CD19^−^ PHA^+^/PHA^−^	r = 0.128 *p* = 0.724	r = 0.809 ***p* = 0.003**	r = −0.709 ***p* = 0.015**	r = −0.827 ***p* = 0.002**	r = −0.155 *p* = 0.650
CD3^−^CD19^+^ PHA^−^	r = 0.074 *p*= 0.820	r = −0.350 *p* = 0.265	r = 0.236 *p* = 0.484	r = 0.049 *p* = 0.880	r = 0.452 *p* = 0.140
CD3^−^CD19^+^ PHA^+^	r = −0.577 *p* = 0.063	r = 0.455 *p* = 0.160	r = −0.545 *p* = 0.083	r = −0.727 ***p* = 0.011**	r = 0.055 *p* = 0.873
CD3^−^CD19^+^ PHA^+^/PHA^−^	r = −0.629 ***p* = 0.028**	r = 0.720 ***p* = 0.008**	r = −0.745 ***p* = 0.008**	r = −0.552 *p* = 0.063	r = −0.434 *p* = 0.158
CD3^−^CD19^−^ PHA^−^	r = −0.269 *p* = 0.423	r = −0.100 *p* = 0.770	r = −0.042 *p* = 0.907	r = 0.182 *p* = 0.593	r = 0.018 *p* = 0.958
CD3^−^CD19^−^ PHA^+^	r = −0.696 ***p* = 0.012**	r = 0.217 *p* = 0.499	r = −0.518 *p* = 0.102	r = −0.350 *p* = 0.265	r = −0.256 *p* = 0.422
CD3^−^CD19^−^ PHA^+^/PHA^−^	r = −0.330 *p* = 0.294	r = 0.764 ***p* = 0.006**	r = −0.54 *p* = 0.071	r = −0.427 *p* = 0.167	r = −0.371 *p* = 0.235

## Data Availability

The original data presented in the study are openly available in Repisalud at https://repisalud.isciii.es/home.

## Supplementary Materials

The following supporting information can be downloaded at: https://www.mdpi.com/article/10.3390/ijms26073351/s1.

## Abbreviations

The following abbreviations are used in this manuscript:

+6M	Six or more months after the establishment SGLT2i treatment
3M	Three months after the establishment SGLT2i treatment
CKD	Chronic kidney disease
CVD	Cardiovascular disease
DBP	Diastolic blood pressures
DMSO	Dimethylsulfoxide
ECAR	Extracellular acidification rate
ETC	Electron transport chain
eGFR	Glomerular filtration rate
FCCP	Carbonylcyanide-p-trifluoromethoxyphenylhydrazone
PBMCs	Peripheral blood mononuclear cells
PHA	Phytohemagglutinin
PRE	Before the establishment SGLT2i treatment
OCR	Oxygen consumption rate
OXPHOS	Oxidative phosphorylation
ROS	Reactive oxygen species
SD	Standard deviation
SBP	Systolic blood pressures
SGLT2	Sodium-glucose co-transporter 2
SGLT2i	Sodium-glucose co-transporter 2 inhibitors
T2D	Type 2 diabetes
TMRM	Tetramethylrhodamine
