# Supplementary material for: Mitochondrial Changes Induced by SGLT2i in Lymphocytes from Diabetic Kidney Transplant Recipients: A Pilot Study
Source: Int J Mol Sci. 2025 Apr 3;26(7):3351. doi: 10.3390/ijms26073351 (PMC11989945; doi:10.3390/ijms26073351)
Supplement: Supplementary file 1 [file ijms-26-03351-s001.zip › ijms-3474921-supplementary.pdf]

Supplementary Material

Treatment with SGLT2 inhibitors induces mitochondrial changes in lymphocytes from kidney transplant recipients with Type 2 Diabetes Mellitus

Isabel Pérez-Flores [1,+], Andrea R. López-Pastor [2,3,+], Ulises Gómez-Pinedo [4], Andrea Gómez-Infantes [2], Laura Espino-Paisán [2], Natividad Calvo Romero [1] , M. Angeles Moreno de la Higuera [1] , Beatriz Rodríguez-Cubillo [1] , Irene Gómez-Delgado [2,3,*], Ana I. Sánchez-Fructuoso [1,5,#] and Elena Urcelay [2,3,#]

*Correspondence: igomezd@salud.madrid.org

Supplementary Tables and Figures

Table S1. Checklist of items that should be included in reports of *cohort studies* by following STROBE Statement.

	Item No	Recommendation	Page(s)
**Title and abstract**	1	(*a*) Indicate the study's design with a commonly used term in the title or the abstract	1
		(*b*) Provide in the abstract an informative and balanced summary of what was done and what was found	1
**Introduction**			
Background/rationale	2	Explain the scientific background and rationale for the investigation being reported	2
Objectives	3	State specific objectives, including any prespecified hypotheses	2-3
**Methods**			
Study design	4	Present key elements of study design early in the paper	12
Setting	5	Describe the setting, locations, and relevant dates, including periods of recruitment, exposure, follow-up, and data collection	12
Participants	6	(*a*) Give the eligibility criteria, and the sources and methods of selection of participants. Describe methods of follow-up	12

		(*b*) For matched studies, give matching criteria and number of exposed and unexposed	NA
Variables	7	Clearly define all outcomes, exposures, predictors, potential confounders, and effect modifiers. Give diagnostic criteria, if applicable	3, 12-14
Data sources/ measurement	8*	For each variable of interest, give sources of data and details of methods of assessment (measurement). Describe comparability of assessment methods if there is more than one group	12-14
Bias	9	Describe any efforts to address potential sources of bias	NA
Study size	10	Explain how the study size was arrived at	12
Quantitative variables	11	Explain how quantitative variables were handled in the analyses. If applicable, describe which groupings were chosen and why	14
Statistical methods	12	(*a*) Describe all statistical methods, including those used to control for confounding	14
		(*b*) Describe any methods used to examine subgroups and interactions	14
		(*c*) Explain how missing data were addressed	14
		(*d*) If applicable, explain how loss to follow-up was addressed	14
		(*e*) Describe any sensitivity analyses	14
**Results**			
Participants	13*	(a) Report numbers of individuals at each stage of study—eg numbers potentially eligible, examined for eligibility, confirmed eligible, included in the study, completing follow-up, and analysed	3, 12
		(b) Give reasons for non-participation at each stage	3, 12
		(c) Consider use of a flow diagram	NA
Descriptive data	14*	(a) Give characteristics of study participants (eg demographic, clinical, social) and information on exposures and potential confounders	3
		(b) Indicate number of participants with missing data for each variable of interest	14

		(c) Summarise follow-up time (eg, average and total amount)	3
Outcome data	15*	Report numbers of outcome events or summary measures over time	3-10
Main results	16	(*a*) Give unadjusted estimates and, if applicable, confounder-adjusted estimates and their precision (eg, 95% confidence interval). Make clear which confounders were adjusted for and why they were included	3-10
		(*b*) Report category boundaries when continuous variables were categorized	NA
		(*c*) If relevant, consider translating estimates of relative risk into absolute risk for a meaningful time period	NA
Other analyses	17	Report other analyses done—eg analyses of subgroups and interactions, and sensitivity analyses	3-10
**Discussion**			
Key results	18	Summarise key results with reference to study objectives	12
Limitations	19	Discuss limitations of the study, taking into account sources of potential bias or imprecision. Discuss both direction and magnitude of any potential bias	11-12
Interpretation	20	Give a cautious overall interpretation of results considering objectives, limitations, multiplicity of analyses, results from similar studies, and other relevant evidence	10-12
Generalisability	21	Discuss the generalisability (external validity) of the study results	11-12
**Other information**			
Funding	22	Give the source of funding and the role of the funders for the present study and, if applicable, for the original study on which the present article is based	14

*Give information separately for exposed and unexposed groups.

Table S2. Primary antibodies used for flow cytometry analyses.

Antibody	Clone	Fluorochrome	Supplier (ref.)
CD3	UCHT1	APC/Fire™	BioLegend (300470)
CD3	HIT3a	PE	BioLegend (300308)
CD19	HIB19	APC	BioLegend (302212)
CD19	HIB19	PB	BioLegend (302232)
Anexin V	-	FITC	BioLegend (640906)

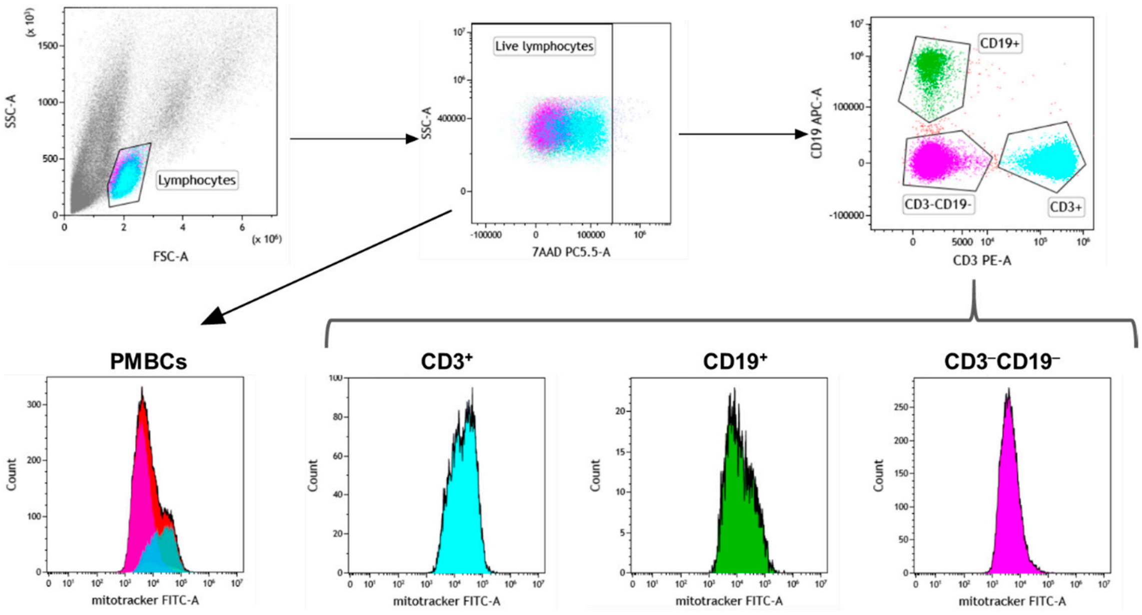

Figure S1. Gating strategies of flow cytometry analyses to assess the mitochondrial mass. 7-AAD was used to exclude non-viable cells. Viable cells were gated according to surface markers: $CD3^{+}CD19^{-}$ (T lymphocytes), $CD3^{-}$ $CD20^{+}$ (B lymphocytes), and $CD3^{-}$ $CD20^{-}$ (mostly NK cells). The median fluorescence intensity (MFI) of the Mitotracker™ Green FM signal was measured for each lymphocyte subpopulation.

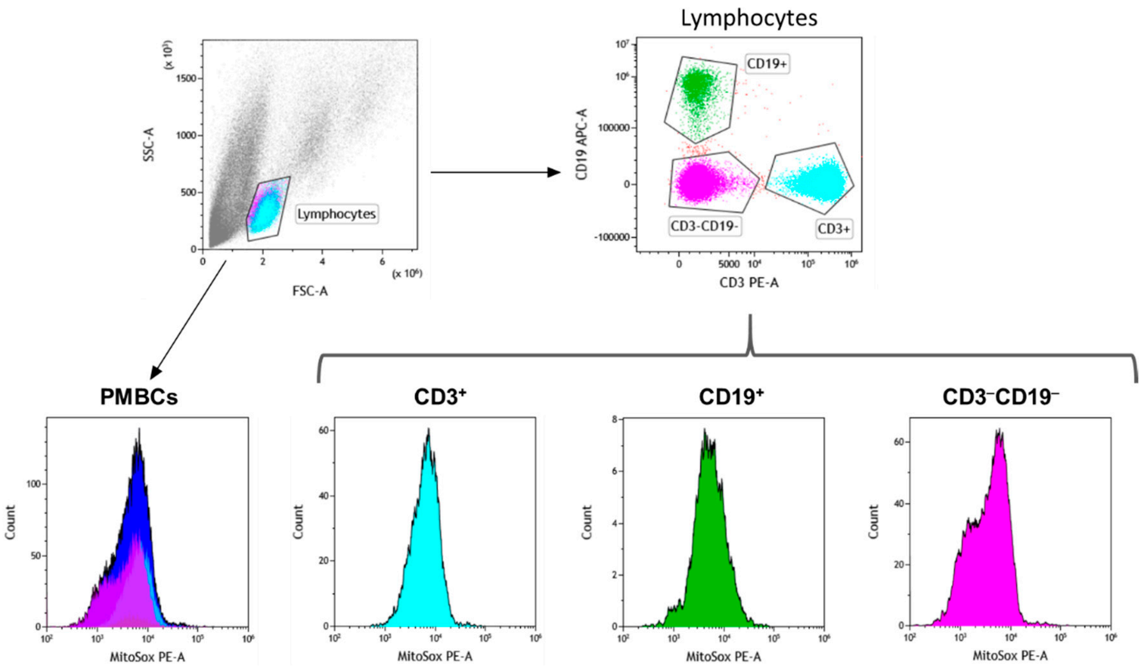

Figure S2. Gating strategies of flow cytometry analyses to assess the mitochondrial superoxide content. Cells were gated according to surface markers: $CD3^{+}CD19^{-}$ (T lymphocytes), $CD3^{-}$ $CD20^{+}$ (B lymphocytes), and $CD3^{-}$ $CD20^{-}$ (mostly NK cells). The median fluorescence intensity (MFI) of the MitoSOX™ Red reagent was measured for each lymphocyte subpopulation.

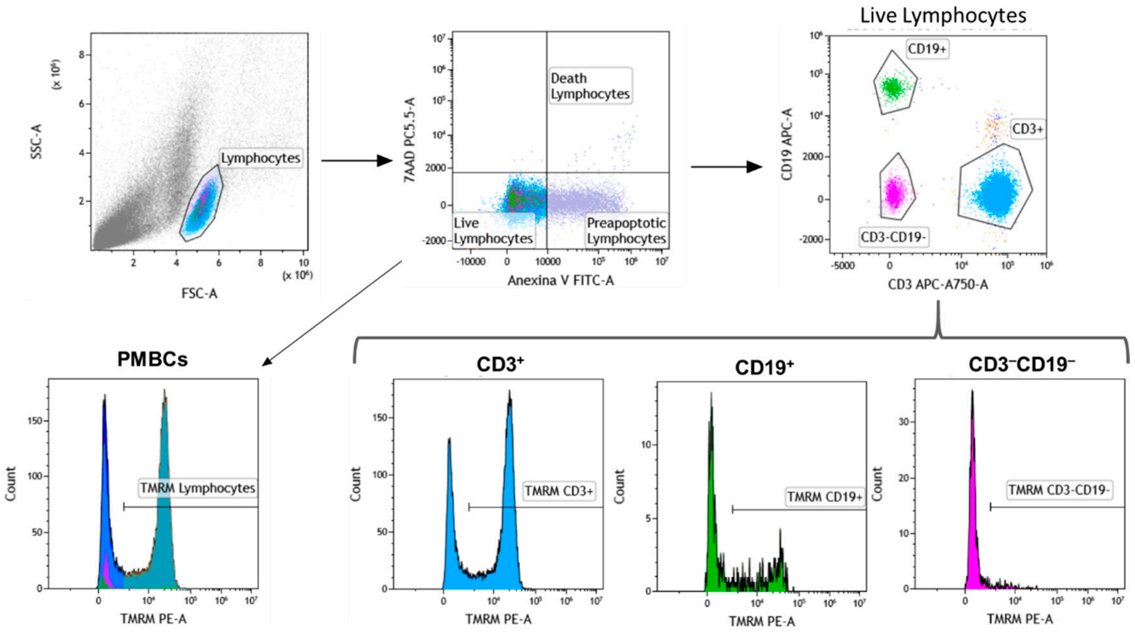

Figure S3. Gating strategies of flow cytometry analyses to assess the mitochondrial membrane potential changes. 7-AAD and Anexin V were used to exclude non-viable cells and preapoptotic cells. Viable cells were gated according to surface markers:

$CD3^+CD19^-$ (T lymphocytes), $CD3^-$ $CD20^+$ (B lymphocytes), and $CD3^-$ $CD20^-$ (mostly NK cells). The median fluorescence intensity (MFI) of the MitoProbe™ TMRM signal was measured for each lymphocyte subpopulation.
